# Epigenetic regulation of nitric oxide synthase 2, inducible (*Nos2*) by NLRC4 inflammasomes involves PARP1 cleavage

**DOI:** 10.1038/srep41686

**Published:** 2017-02-02

**Authors:** Carina de Lima Buzzo, Tiago Medina, Laura M. Branco, Silvia L. Lage, Luís Carlos de Souza Ferreira, Gustavo P. Amarante-Mendes, Michael O. Hottiger, Daniel D. De Carvalho, Karina R. Bortoluci

**Affiliations:** 1 Centro de Terapia Celular e Molecular (CTC-Mol) e Departamento de Ciências Biológicas - Universidade Federal de São Paulo, São Paulo, Brazil; 2 Departamento de Microbiologia, Instituto de Ciências Biomédicas, Universidade de São Paulo, São Paulo, Brazil; 3 Princess Margaret Cancer Centre, University Health Network, Toronto, ON, M5G 2M9, Canada; 4 Instituto de Ciências Biomédicas, Universidade de São Paulo, São Paulo and Instituto de Investigação em Imunologia, Instituto Nacional de Ciência e Tecnologia (INCT-iii), Brazil; 5 Department of Molecular Mechanisms of Disease, University of Zurich, Zurich, Switzerland; 6 Department of Medical Biophysics, University of Toronto, Toronto, ON, M5G 2M9, Canada

## Abstract

Nitric oxide synthase 2, inducible (*Nos2*) expression is necessary for the microbicidal activity of macrophages. However, NOS2 over-activation causes multiple inflammatory disorders, suggesting a tight gene regulation is necessary. Using cytosolic flagellin as a model for inflammasome-dependent NOS2 activation, we discovered a surprising new role for NLRC4/caspase-1 axis in regulating chromatin accessibility of the *Nos2* promoter. We found that activation of two independent mechanisms is necessary for NOS2 expression by cytosolic flagellin: caspase-1 and NF-κB activation. NF-κB activation was necessary, but not sufficient, for NOS2 expression. Conversely, caspase-1 was necessary for NOS2 expression, but dispensable for NF-κB activation, indicating that this protease acts downstream NF-κB activation. We demonstrated that epigenetic regulation of *Nos2* by caspase-1 involves cleavage of the chromatin regulator PARP1 (also known as ARTD1) and chromatin accessibility of the NF-κB binding sites located at the *Nos2* promoter. Remarkably, caspase-1-mediated *Nos2* transcription and NO production contribute to the resistance of macrophages to *Salmonella typhimurium* infection. Our results uncover the molecular mechanism behind the constricted regulation of *Nos2* expression and open new therapeutic opportunities based on epigenetic activities of caspase-1 against infectious and inflammatory diseases.

Innate immune responses rely on Pattern Recognition Receptor (PRR) families responsible for the activation of redundant and non-redundant effector responses during infectious and non-infectious degenerative processes. Inflammasomes are cytosolic platforms composed of members from the nucleotide-binding oligomerization domain (NOD), leucine-rich repeat (LRR)-containing protein (NLR) or the pyrin and HIN domain-containing protein (PYHIN) families responsible for the recruitment and activation of inflammatory caspase-1 and caspase-11 (caspase-4 in humans), respectively^1^,^2^. The complexes containing NLRP1, NLRP3, NLRC4 from NLR family and absent in melanoma 2 (AIM2) and pyrin from PYHIN family comprise the best-characterized inflammasomes. These platforms are assembled in response to a wide range of pathogen-associated molecular patterns (PAMPs), damage-associated molecular patterns (DAMPs) or cytosolic disturbances^2^. These stimuli induce conformational changes in NLR or PYHIN-containing proteins, ultimately leading to recruitment of caspase-1/11 through homotypical interactions, which may or may not involve the participation of the adaptor molecule ASC (apoptosis-associated speck-like protein).

Inflammasomes are central players in a series of infectious diseases and inflammatory processes. However, the precise molecular mechanisms involved in the regulation of inflammasomes activation remain to be elucidated^3^,^4^. The major effector mechanisms mediated by caspase-1 is the maturation and secretion of pro-inflammatory cytokines IL-1β and IL-18 and the induction of a cell death process named pyroptosis. These mechanisms contribute to control infections by inducing strong inflammatory response and recruitment of effector cells, which lead to rapid elimination of pathogens replication foci^2^,^5^.

In the last years, additional effector mechanisms mediated by inflammasomes have been described, such as the secretion of inflammatory mediators, activation of adaptive immune responses and induction of macrophage microbicidal activities (Reviewed by ref. 6). Interestingly, many of these responses are shared with other PRRs, particularly the transmembrane Toll-like receptors (TLR), including the activation of Nitric Oxide Synthase 2, inducible (NOS2). *Nos2* expression, and the consequent production of nitric oxide (NO), represents an important microbicidal mechanism exploited by the innate immune system. Defects in *Nos2* expression have been associated with enhanced susceptibility to a variety of infectious diseases^7^. On the other hand, uncontrolled expression of *Nos2* may lead to the induction of different inflammatory pathologies, such as neurological disorders, liver dysfunctions, atherosclerosis, sepsis and tumors^8^,^9^,^10^. Thus, activation of inflammasomes, as well as the transcriptional activation of *Nos2* during infection, must be tightly regulated.

We previously demonstrated that activation of NAIP5/NLRC4 inflammasomes by cytosolic flagellin, the structural protein of flagella expressed by motile bacteria, leads to NOS2 activation^11^. More relevantly, we showed that the caspase1/11-induced *Nos2* expression plays a key role in the control of intracellular infections^11^,^12^. However, the precise molecular mechanism of how inflammassome activation regulates *Nos2* expression remains to be elucidated.

The promoter region of the mouse and human *Nos2* gene contains binding sites for several transcription factors^13^,^14^,^15^,^16^. However, the induction of *Nos2* through inflammatory pathways, such as TLR and IL-1R is mainly dependent on nuclear factor kappaB (NF-κB) (Reviewed by ref. 17). Intriguingly, inflammasome-induced *Nos2* activation occurs independently of IL-1β and IL-18, but requires caspase-1 for its transcriptional regulation^11^, suggesting a novel role of caspase-1 in the regulation of gene transcription.

In fact, here we demonstrated a key role for caspase-1 in regulating chromatin accessibility at the *Nos2* gene promoter, allowing NF-κB binding and gene expression upon inflammasome activation, an event that involves the cleavage of poly(ADP-ribose) polymerase-1 (PARP1, also known as ADP-ribosyltransferase diphtheria like 1 (ARTD1)). This novel molecular mechanism of gene expression mediated by caspase-1 has a significant impact on the microbicidal capacity of macrophages and may be exploited as an important new avenue for drug discovery and future therapeutic interventions to a variety of diseases related to inflammasome activation.

## Results

### Activation of NLRC4 inflammasomes by cytosolic flagellin induces NOS2 expression independently of MyD88

**Figure 1:**
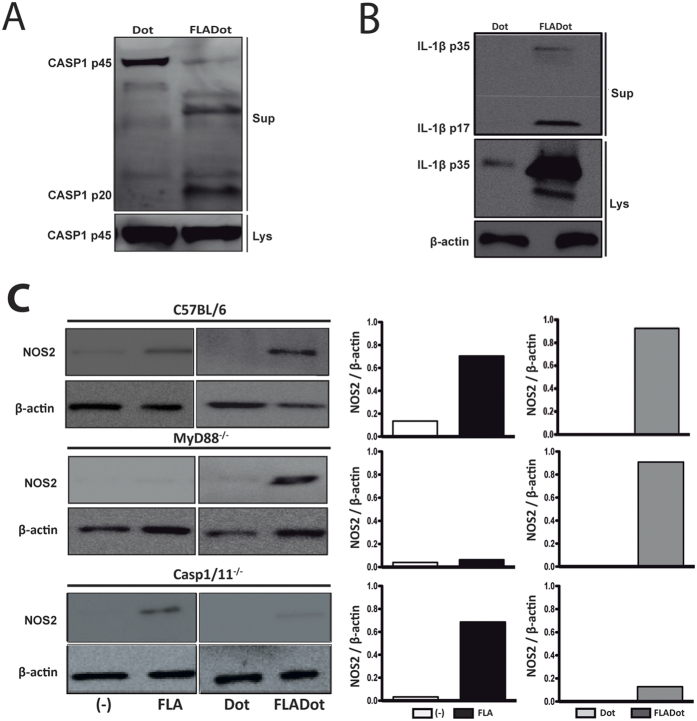
Cytosolic flagellin induces NOS2 expression in a Myd88-independent and casp1/11-dependent manner. Starch-elicited peritoneal macrophages (PMs) isolated from C57BL/6 WT mice were stimulated with flagellin from *Bacillus subtilis* inserted on DOTAP (Dot) (FLADot) [3 μg/mL] for 6 hr. (**A**) Caspase-1 activation (p20) and (**B**) mature IL-1β secretion (p17) were assessed by western blotting in total cell extracts and supernatants. Cells were stimulated with lipid vesicle empty (Dot) as negative control. (**C**) PMs from C57BL/6 WT, casp1/11^−/−^ and MyD88^−/−^ mice were stimulated with Dot, FLA [3 μg/mL] or FLADot [3 μg/mL] for 24 hr and NOS2 expression was analyzed by western blotting in total cell extracts. Western blot densitometric analysis was performed using ImageJ software and Nos2 relative expression was normalized according to β-actin expression. Data are representative from at least three independent experiments.

Cytosolic flagellin is known to activate NAIP5/NLRC4 inflammasomes, leading to caspase-1 cleavage, secretion of mature IL-1β, pyroptosis and *Nos2* expression (Reviewed by ref. 6). Indeed, purified flagellin from *Bacillus subtillis* inserted into lipid vesicles (FLADot), which allow its delivery to the cell cytosol, induced caspase-1 activation (Fig. 1A) and secretion of mature IL-1β by macrophages from C57BL/6 wild-type (WT) mice (Fig. 1B). Empty lipidic vesicles or flagellin alone (a known TLR5 agonist) did not induce caspase-1 activation or secretion of mature IL-1β (Fig. 1A,B and Supplemental Figure 1A). Moreover, induction of IL-1β secretion by cytosolic flagellin was abolished in macrophages derived from NLRC4^−/−^ mice (Supplemental Figure 1A). In addition, as we previously described^11^, cytosolic flagellin (FLADot) was able to induce NOS2 expression (Fig. 1C), which was lost in macrophages from caspase-1/11^−/−^ (casp1/11^−/−^) but not in MyD88^−/−^ cells (Fig. 1C). Conversely, NOS2 induced by free flagellin (FLA) did not require caspase-1/11 and occurred in a MyD88-dependent manner (Fig. 1C), suggesting the existence of two independent pathways for the induction of *Nos2* in response to flagellin. Since MyD88 is a key adapter molecule necessary to transduce the signal from TLR5, IL1R and IL18R^18^,^19^, our data confirm the activation of NLRC4 inflammasomes by cytosolic flagellin induces NOS2 expression that is likely independent of TLR5, IL-1β and IL-18 pathways.

### NF-κB activation is required for NLRC4 inflammasome-induced NOS2 expression in response to cytosolic flagellin

**Figure 2:**
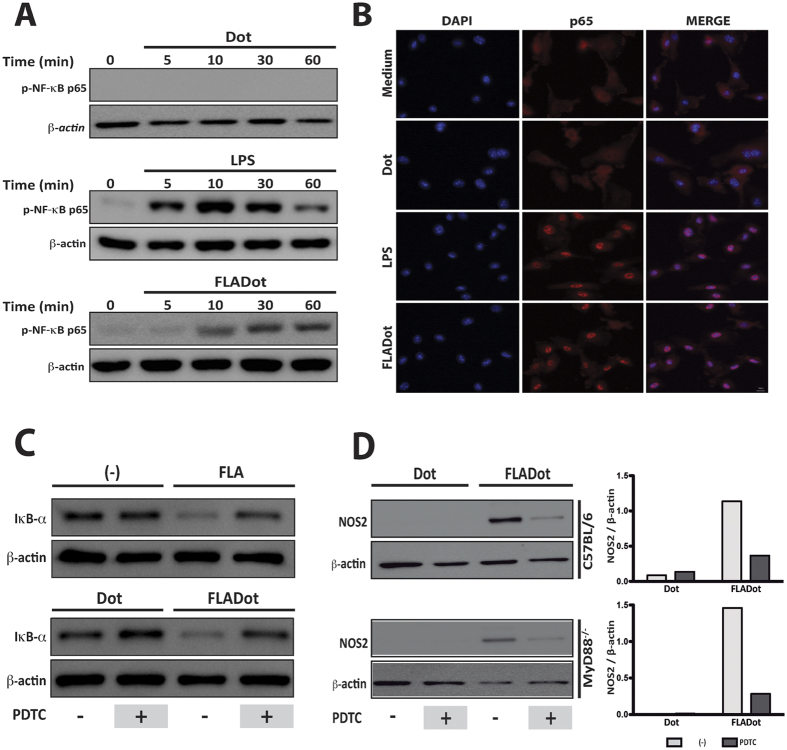
Cytosolic flagellin induces NF-κB activation that is required for NOS2 expression. (**A**) PMs from C57BL/6 WT mice were stimulated with Dot, FLADot [3 μg/mL] or LPS [1 μg/mL] as indicated for 5 to 60 minutes and the phospho p65-NF-κB subunit was analyzed by western blotting. (**B**) BMDMs from C57BL/6 WT mice were stimulated with Dot, LPS [100 ng/mL] or FLADot [3 μg/mL] for 30 min and p65-NF-κB nuclear translocation was assessed by immunofluorescence microscopy. (**C**) IκB-α degradation was assessed by western blotting 30 min after stimulations. (**D**) PMs from WT and MyD88^−/−^ mice were stimulated with FLADot [3 μg/mL] in the presence or absence of PDTC for 24 hr. Total cell extract was prepared and NOS2 were analyzed by western blotting. Western blot densitometric analysis was performed using ImageJ software and NOS2 relative expression was normalized according to β-actin expression. Data are representative from at least three independent experiments.

The induction of NOS2 expression by TLRs or cytokine receptors is a well-described process that depends on NF-κB activation^20^,^21^,^22^,^23^. Our previous findings demonstrate the requirement of caspase-1 for *Nos2* gene and protein expression upon stimulation of macrophages with cytosolic flagellin^11^. However, the molecular mechanism involved in the activation of *Nos2* by cytosolic receptors remains to be dissected. Here, we found that similarly to TLR agonists (free flagellin or LPS), cytosolic flagellin was also able to induce NF-κB activation, as demonstrated by p65 phosphorylation (Fig. 2A), its nuclear translocation (Fig. 2B) and IκB-α degradation (Fig. 2C). To confirm that NF-κB activation is required for cytosolic flagellin-induced NOS2 expression, we used the NF-κB inhibitor PDTC (Pyrrolidinedithiocarbamate). PDTC inhibits NF-κB activation by preventing IκB-α phosphorylation and degradation (Fig. 2C)^24^. Indeed, NOS2 expression by cytosolic flagellin was abrogated in WT as well as in MyD88^−/−^ macrophages in the presence of PDTC (Fig. 2D). Altogether, these data indicate that NF-κB is necessary for cytosolic flagellin-induced NOS2 expression.

### Caspase-1 is not required for NF-κB activation but necessary for Nos2 activation in response to flagellin

**Figure 3:**
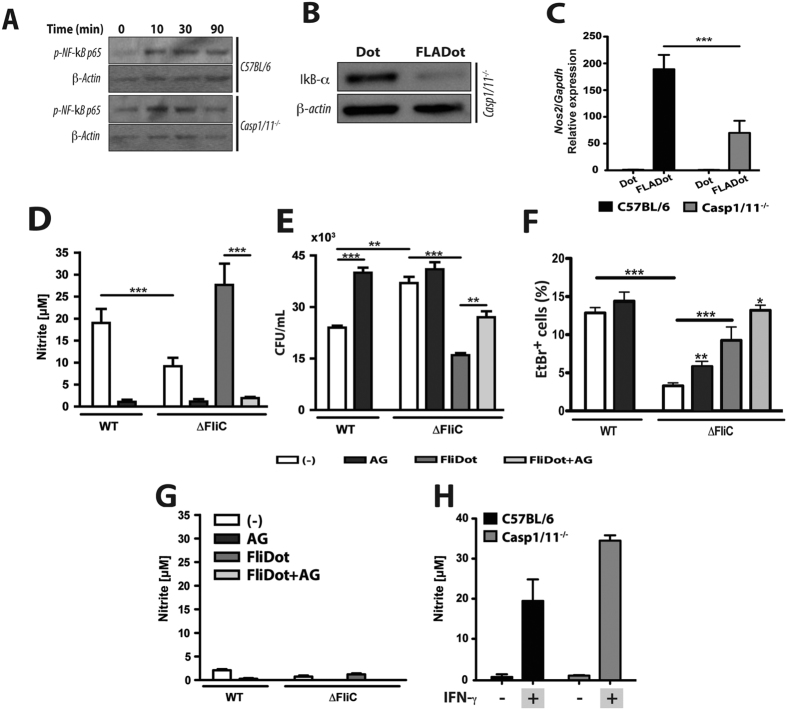
Caspase-1/11-dependent NOS2 activation is involved in the control of *S. Typhimurium* infection by macrophages. (**A**) PMs from C57BL/6 and casp1/11^−/−^ mice were stimulated with Dot or FLADot [3 μg/mL] for indicated times and the phospho p65-NF-κB subunit was analyzed by western blotting. (**B**) IκB-α degradation was assessed by western blotting in PMs from casp1/11^−/−^ mice 30 minutes after Dot and FLADot stimulations. (**C**) Bone marrow derived macrophages (BMDM) from C57BL/6 and casp1/11^−/−^ mice were stimulated with Dot or FLADot [3 μg/mL] for 3–4 hr and mRNA levels were determined by real-time RT-PCR. Samples were normalized to GAPDH expression levels. (**D**) PMs from C57BL/6 or (**G**) casp1/11^−/−^ mice were infected with the flagellin-suficient *S.Typhimurium* 1412 strain (WT) or the flagellin deficient *S. Typhimurium* 2157 strain – ΔFliC) (3 × 10^4^ bacteria per 3 × 10^5^ macrophages) for 1 hr in the presence or absence of aminoguanidine (AG). After infection, cultures were treated with gentamicine [50 μg/ml] for 50 min to eliminate extracellular bacterial and maintained with 5 μg/ml of gentamicin for 24–48 hr in the presence of purified *S. Tyhimurium-*derived flagellin inserted into DOTAP (FliDot) [1 μg/mL] as indicated. NO production was assessed by Griess methods in culture supernatants after 48 hr. (**E**) CFU counting from C57BL/6 PMs infected with WT or ΔFliC *S. Typhimurium* after 24 hr. (**F**) Cytotoxicity was assessed as the percentage of EtBr single-positive cells in fluorescence micrographs according to AO/EtBr staining after 24 hr of infection. Numbers represent the means ± SEM of at least 16 images per treatment. (**H**) PMs from C57BL/6 WT and casp1/11^−/−^ mice were stimulated with (−) or rIFN-γ [2,5 ng/mL] for 24 hr and NO production was assessed by Griess methods in culture supernatants. Bars represent the mean S.D. of triplicate samples. Data are representative of three independent experiments. **p > 0.01; ***p < 0.001.

Since NOS2 induced by cytosolic flagellin required both caspase-1 and NF-κB, we hypothesize that caspase-1 acts through NF-κB to induce NOS2 expression. Surprisingly, we observed that even in the absence of caspase-1, cytosolic flagellin was still able to activate NF-κB (Fig. 3A,B). Macrophages derived from WT and casp1/11^−/−^ showed similar kinetics of p65 phosphorylation, which was detectable by 10 minutes after cytosolic flagellin stimulation (Fig. 3A). Similarly to WT cells (Fig. 2B), IκB-α degradation was also observed in macrophages from casp1/11^−/−^ mice, 30 minutes after cytosolic flagellin stimulation (Fig. 3B).

Although caspase-1 was dispensable for NF-κB activation, it was required for robust *Nos2* expression and activation. Macrophages derived from casp-1/11^−/−^ mice (and cells treated with z-YVAD-fmk^11^) showed impaired expression of NOS2, both at the protein (Fig. 1C) and mRNA (Fig. 3C) levels, upon stimulation with cytosolic flagellin. Accordingly, macrophages from WT mice (Fig. 3D), but not from casp1/11^−/−^ mice (Fig. 3G), were able to secrete high levels of NO in response to the infection with the intracellular bacteria *Salmonella typhimurium (S. typhimurium*). Lower levels of NO was observed in response to flagellin-deficient (ΔFliC) mutant *S. typhimurium* compared to wild type bacteria (Fig. 3D), which correlated with the higher CFU numbers recovered from these cultures (Fig. 3E). The addition of AG, a pharmacological inhibitor of NOS2, significantly increased the number of CFU observed in cultures infected with wild type but not ΔFliC *S. typhimurium* (Fig. 3E), suggesting a role for cytosolic flagellin-mediated NO secretion to the control of *S. typhimurium* by macrophages. To further confirm the requirement of flagellin we added cytosolic flagellin to these macrophages infected with ΔFliC *S. typhimurium*. As expected, cytosolic flagellin induced an increase in the NO secretion (Fig. 3D), which resulted in the better control of infection (Fig. 3E). Again, this effect was reverted by the addition of AG (Fig. 3E), demonstrating that the non-canonical induction of *Nos2* via cytosolic flagellin is important to the control of *S. typhimurium* infection.

As expect, in comparison to ΔFliC *S. typhimurium,* wild type *S. typhimurium* also induced higher frequencies of cells that incorporated Ethidium Bromide (EtBr) and lost the staining for Acridine Orange (AO) (Fig. 3F), a phenomenon consistent with the induction of pyroptosis^25^. Similar as observed for NO production, the addition of purified cytosolic flagellin resulted in increased cell death in ΔFliC *S. typhimurium*-infected macrophages. Importantly, AG did not inhibit cell death in any situation (Fig. 3F). Conversely, a slight higher frequencies in cell death was observed in the presence of AG during infection with ΔFliC *S. typhimurium* (Fig. 3F), which could be explained by the increased numbers of CFU (Fig. 3E), since AG had no effect on cell death in the absence of infection (data not shown) or during infection with wild type *S. typhimurium* (Fig. 3F). These results demonstrate that flagellin-induced NO secretion acts through a non-redundant manner with pyroptosis to optimize the control of *S. typhimurium* by macrophages.

Importantly, although cytosolic flagellin is also able to induce a caspase 1/11-independent inflammatory form of cell death^25^, NO secretion was not observed in macrophages from casp1/11^−/−^ in all above situations (Fig. 3G), confirming the relevance of the caspase-1/11-induced NOS2 activation to the control of *S. typhimurium* by macrophages. Notably, casp1/11^−/−^ macrophages were fully able to secrete NO in response to rIFN-γ plus LPS (Fig. 3H), thus indicating these cells have no intrinsic defect in *Nos2* processing. Altogether, these results suggest that caspase-1 is dispensable for the activation of NF-κB, however, its induction is necessary for robust NOS2 activation in response to cytosolic flagellin.

### PARP1 cleavage is required for cytosolic flagellin-induced Nos2 expression

**Figure 4:**
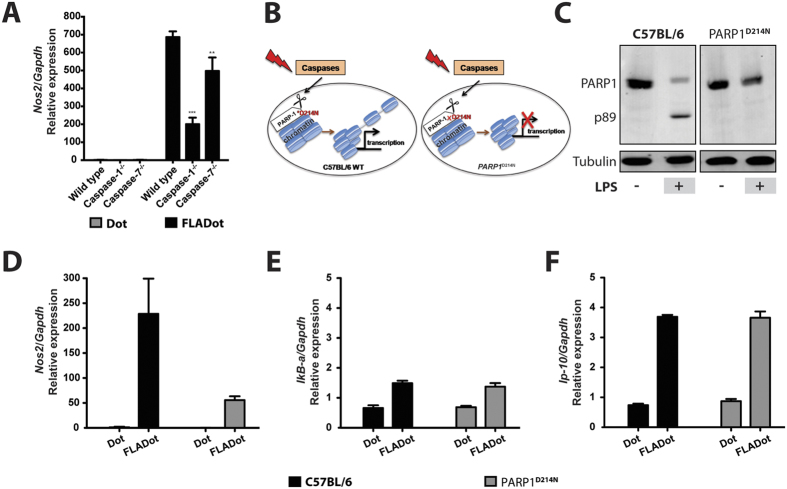
NLRC4 inflammasome-induced *Nos2* expression in response to cytosolic flagellin requires PARP1 cleavage at D214N site. (**A**) Bone marrow derived macrophages (BMDM) from C57BL/6, casp1/11^−/−^ and casp7^−/−^ mice were stimulated with Dot or FLADot [3 μg/mL] for 3–4 hr and mRNA levels were determined by real-time RT-PCR. Samples were normalized to GAPDH expression levels. (**B**) Schematic representation of the point mutation in the cleavage site for caspases in the PARP1 molecule present in PARP1^D214N^ mice and its consequence for chromatin modulation. (**C**) BMDM isolated from C57BL/6 WT and PARP1^D214N^ mice were stimulated with LPS [100 ng/mL] for 6 hr. Total cell extracts were prepared, and PARP1 cleavage was analyzed by western blotting. BMDM from C57BL/6 WT and PARP1^D214N^ mice were stimulated with FLADot [3 μg/mL] for 3–4 hr and mRNA levels of *No*s2 (**D**), IκB-α (**E**) and IP-10 (**F**) were determined by real-time RT-PCR analysis. WT and PARP1^D214N^ samples were normalized to their GAPDH expression levels and expressed as relative mRNA levels. Bars represent the mean ± SEM of triplicate samples. Data are representative of three (**A** and **C**) or five (**D**–**F**) independent experiments. **p > 0.01; ***p < 0.001.

Up to this point, our data demonstrated that NF-κB activation is necessary but not sufficient for *Nos2* expression in response to cytosolic flagellin. Moreover, our data suggested that caspase-1 is necessary for *Nos2* expression downstream of NF-κB activation. Thus, we hypothesize that caspase-1 may play a role in regulating the chromatin accessibility for NF-κB binding to the regulatory elements of the *Nos2* promoter. Corroborating our hypothesis, recently published work demonstrated that caspase-7 activation by LPS can lead, in a non-apoptotic manner, to PARP1 cleavage, resulting in chromatin decondensation, which promotes the transcription of a specific set of NF-κB target genes, such as *Csf2, Il-6* and *Lif* but not *Ip-10*^26^. However, in response to cytosolic flagellin, caspase-1/11 seems to play a central role in promoting the transcription of *Nos2*, since casp7^−/−^ macrophages present only a minor defect in the induction of *Nos2* expression in comparison to casp1/11^−/−^ cells (Fig. 4A).

To evaluate the involvement of PARP1 cleavage in the transcriptional activation of *Nos2* by cytosolic flagellin, we took advantage of knockin PARP1^D214N^ mice (Fig. 4B), in which PARP-1 is uncleavable even in the presence of a highly inflammatory stimulus, such as LPS (Fig. 4C). Interestingly, macrophages derived from PARP1^D214N^ mice fail to robustly up-regulate *Nos2* after cytosolic flagellin stimulation (Fig. 4D). Importantly, the requirement of PARP1 cleavage seems to be specific for the *Nos2* gene since the transcription of other genes, such as *I*κ*B-α* (Fig. 4E) and *Ip-10* (Fig. 4F), were not affected after exposure of PARP1^D214N^ macrophages to cytosolic flagellin, indicating that NF-κB activation is intact in these cells. These results imply that PARP1 cleavage is required for *Nos2* transcriptional activation in response to cytosolic flagellin, suggesting caspase-1 is regulating the access of NF-κB to its binding sites at the *Nos2* gene promoter region.

### Binding sites of NF-κB in the *Nos2* gene promoter are not accessible in the absence of caspase-1/11

**Figure 5:**
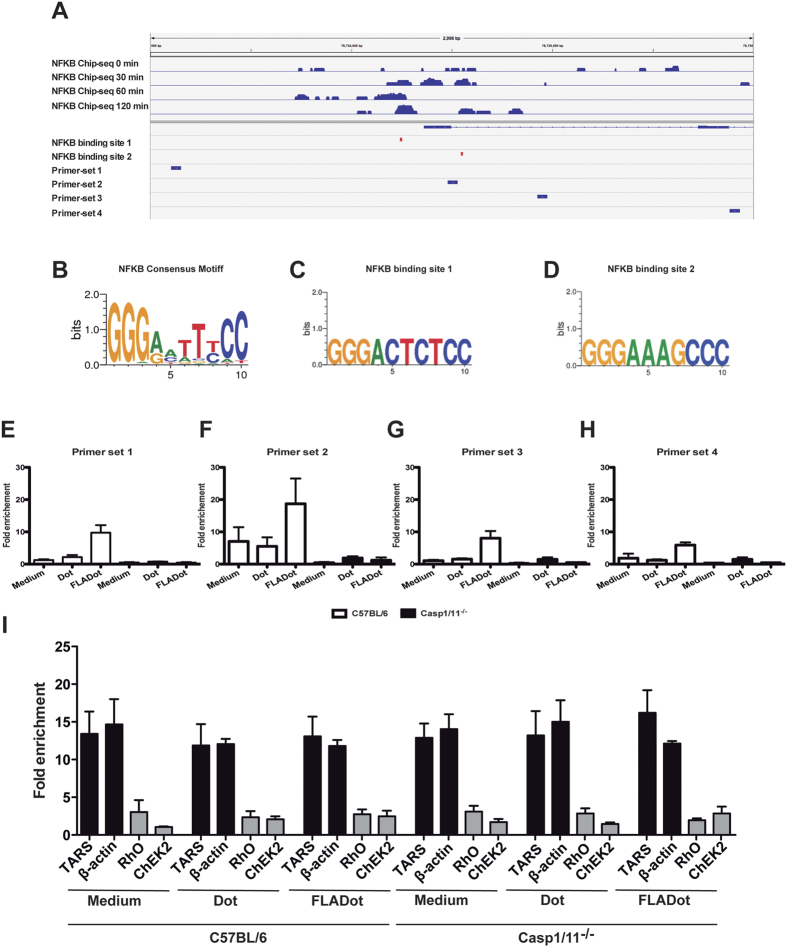
Binding sites of NF-κB at the *Nos2* gene promoter are not accessible in the absence of caspase-1/11. (**A**) Bone marrow-derived dendritic cells (BMDC) were stimulated with LPS [100 ng/ml] for 30–120 min and the enrichment of NF-κB binding to the *Nos2* gene promoter was identified using public available ChIP-seq data. (**A**–**D**) Identification of two putative NF-κB binding sites around the *Nos2* promoter. (**E**–**H**) Bone marrow-derived macrophages (BMDM) from WT or casp1/11^−/−^ were stimulated with Dot or FLADot and after 3 h the chromatin accessibility at the *Nos2* gene promoter was analyzed using Transpoase-Accessible Chromatin Assay (ATAC) coupled with qPCR. (**I**) Positive control of chromatin accessibility in BMDM stimulated as indicated.

Using public available ChIP-seq data for NF-κB in bone marrow-derived dendritic cells (BMDC) stimulated with LPS (GSE36104)^27^, we were able to identify enrichment of NF-κB binding to the *Nos2* gene promoter between 30 to 120 minutes after LPS treatment (Fig. 5A). We performed motif analysis on these NF-κB peaks around the *Nos2* gene promoter and identified two putative NF-κB binding sites (Fig. 5A–D). Next, we used the Assay for Transposase-Accessible Chromatin (ATAC) coupled with qPCR in order to evaluate the accessibility of chromatin around these putative NF-κB binding sites of the *Nos2* gene. We designed one primerset between the two NF-κB binding sites (primerset 2), one primerset upstream (primerset 1) and two primersets downstream (primerset 3 and 4). We observed that cytosolic flagellin robustly increased chromatin accessibility at the *Nos2* gene promoter in BMDM from WT mice, especially in the regions close to the NF-κB binding sites (Fig. 5E–H, white bars). However, in the absence of caspase-1/11, cytosolic flagellin lost its ability to increase chromatin accessibility at the *Nos2* gene promoter (Fig. 5E–H, black bars). These results are unlikely to be caused by global change in chromatin accessibility in the casp1/11^−/−^ cells since we could not observe changes in chromatin accessibility for our positive and negative controls between WT and casp1/11^−/−^ (Fig. 5I). Notably, cytosolic flagellin also failed to increase the accessibility of chromatin at the *Nos2* gene promoter in NLRC4^−/−^ macrophages (Supplemental Figure 1B–E), demonstrating that NLRC4/caspase-1 axis is central in the epigenetic regulation of *Nos2* gene transcription in response to cytosolic flagellin. To sum up, our results describe a key regulatory role of caspase-1 on chromatin accessibility at the *Nos2* gene promoter upon NLRC4 inflammasome activation. This increased chromatin accessibility allows NF-κB binding and transcriptional activation of *Nos2*.

## Discussion

Inflammasomes are immune platforms that lead to caspase-1 activation in response to a wide range of stimuli, including bacterial, protozoan, viral and fungal infections^28^,^29^,^30^,^31^. Active caspase-1 mediates a series of effector responses required for the control of infections. Among these effector responses, expression of *Nos2* through inflammasomes is involved in the control of *Legionella pneumophila*^11^, *Trypanosoma cruzi*^12^, *Leishmania spp*^32^ and *Salmonella Typhimurium* (Fig. 3). However, overactivation of NOS2 can cause tissue damage and consequent inflammatory pathologies such as asthma, cardiovascular and neurological diseases, liver and renal dysfunctions, atherosclerosis, tumors, coagulation disorders, sepsis, among others^8^,^33^,^34^,^35^. Both microbicidal activity and the development of inflammatory pathologies are a result of the key role of NO and peroxynitrite (ONNO^−^), a powerful free radical formed by the reaction of NO with superoxide (O2^−^), in the modification of a wide range of different proteins^8^,^36^,^37^. Therefore, understanding the molecular mechanisms that lead to *Nos2* activation may allow the development of therapeutic strategies aiming to increase its activation in case of infections or reduce its activation in inflammatory pathologies.

**Figure 6:**
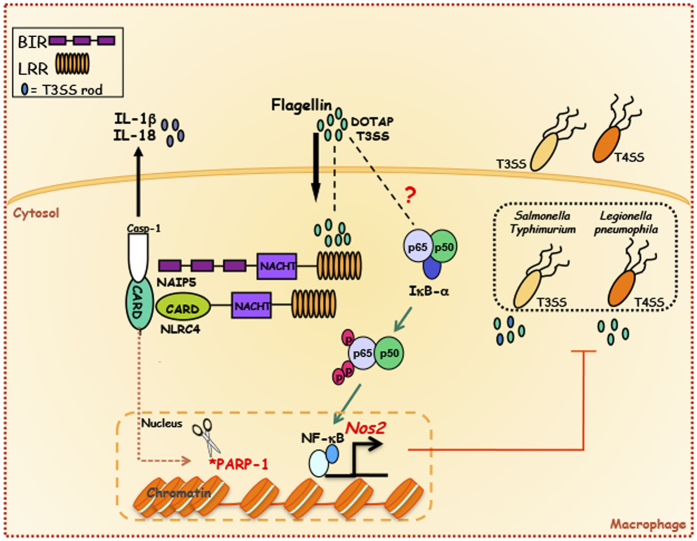
NLRC4 inflammasomes regulate chromatin accessibility at the *Nos2* gene promoter in response to cytosolic flagellin by an event that involves PARP-1 cleavage. Cytosolic flagellin induces NAIP5/NLRC4-caspase-1 pathway, and in parallel, activates NF-κB transcription factor by an unknown mechanism. Inflammasome activation leads to the cleavage of PARP1, which, in its turn, is likely released from the chromatin, resulting in chromatin decondensation and consequently NF-κB-dependent *Nos2* gene transcription. The product of NOS2 expression (nitric oxide) is involved in the control of intracellular replication of pathogens in macrophages.

The induction of *Nos2* expression by TLRs or cytokine receptors is a well-described process that depends on NF-κB activation^20^,^21^,^22^,^23^. However, activation of *Nos2* by cytosolic receptors is significantly less known. Our previous findings demonstrate the requirement of caspase-1 for *Nos2* gene and protein expression upon stimulation of macrophages with cytosolic flagellin^11^. Here we dissected this molecular mechanism and found that robust expression of *Nos2* by stimulation of macrophages with cytosolic flagellin is dependent on two pathways (Fig. 6). First, cytosolic flagellin was able to induce NF-κB, in a process independent of caspase-1 that was required, but not sufficient, for robust *Nos2* expression. Second, activation of the NLRC4 inflammasomes by cytosolic flagellin was able to activate caspase-1, leading to cleavage of PARP1 and subsequent increased chromatin accessibility at the NF-κB binding sites of the *Nos2* promoter.

The exact mechanism by which cytosolic flagellin activates NF-κB remains to be elucidated. However, it does not require caspase-1. Importantly, the inhibition of NF-κB also abrogated NOS2 expression in response to cytosolic flagellin in macrophages from MyD88^−/−^ mice. Since NOS2 activation in response to cytosolic flagellin was preserved in MyD88^−/−^ macrophages, it suggests that the activation of caspase-1/NF-κB axis involved in the induction of *Nos2* occurs independently of TLR5, IL-1R, IL-18R pathways.

Although IL-1β and IL-18 are the best-studied caspase-1 substrates, it is well documented that caspase-1 also cleave less conventional substrates, including PARP1^38^. PARP1 catalyzes the polymerization of ADP-ribose units from donor NAD^+^molecules^39^,^40^. During apoptosis and pyroptosis, PARP1 is cleaved by caspases, resulting in the release of two fragments (24 and 89 kDa) and the inactivation of the enzymatic activity^38^,^41^. Although historically studied in the context of genotoxic stress signaling and apoptosis, PARP1 has recently been linked to the regulation of chromatin structure, transcription, and chromosome organization^26^,^42^,^43^. These findings came from the observations that PARP1^D214N^ mice developed normally, indicating that cleavage of PARP1 is not required during apoptosis^44^ but is more likely required for other important cellular functions. In fact, increasing numbers of studies have provided evidence that PARP1 can regulate gene expression also independently of its enzymatic activity, especially by inducing structural chromatin changes (reviewed by ref. 45). Under steady state conditions, PARP1 maintains chromatin in a condensed state, thus dampening transcriptional activation of genes. However, under inflammatory or stress conditions, PARP1 is cleaved by caspases, which could promote its eviction from the chromatin and subsequently a local chromatin decondensation, leaving the DNA accessible for transcription factors. In this context, Erener *et al*.^26^ demonstrated the cleavage of PARP1 by caspase-7 promote the transcription of a specific set of NF-κB target genes, such as *Csf2, Il-6* and *Lif* but not *Ip-10.* In our system, caspase-1 seems to have a significant higher impact than caspase-7 on *Nos2* expresson, pointing caspase-1 activation and PARP1 cleavage as central events in the regulation of *Nos2* transcription in response to cytosolic flagellin.

Altogether, we provided strong evidence for the molecular mechanism that regulates *Nos2* expression upon inflammassome activation. This mechanism is dependent on NLRC4/caspase-1, PARP1 cleavage, and NF-κB activation. Our data suggests that the increased chromatin accessibility at *Nos2* promoter is caused by caspase-1-dependent cleavage of PARP. However, further experiments are necessary to establish a direct link between PARP cleavage and increased chromatin accessibility at *Nos2* promoter by cytosolic flagelin. Importantly, stimulation of macrophages with cytosolic flagellin, even in the absence of caspase-1/11, does not activate the apoptosis program^25^, which supports the non-apoptotic role of PARP-1 in gene regulation. Similar as found during the activation of NLRC4 inflammasomes by *S. typhimurium*, the activation of NLRP3 with ATP or nigericin also induces the cleavage of PARP1 in a NLRP3, ASC and caspase-1- dependent manner^38^, although it remains to be elucidated the involvement of NLRP3 in the epigenetic regulation of *Nos2.* It is important to note that PARP1 cleavage seems to have only a minor or no effect on pyroptosis and IL-1β secretion, respectively, in response to the activation of NLRP3 and NLRC4 inflammasomes^38^, reinforcing the idea that cell death and epigenetic regulation are independent events mediated by caspases/PARP1 axis.

Remarkably, our data clear demonstrated that the NLRC4 inflammasome-mediated epigenetic regulation of *Nos2* is essential for its full activation and biological properties, such as macrophage microbicidal capacity. It is well known that inflammatory processes of cell death induced by cytosolic flagellin are important NAIP5/NLRC4 inflammasomes-mediated effector mechanisms involved in the control of infections^5^,^25^. The delivery of flagellin into cell cytosol leads to caspase-1/11-dependent and -independent forms of cell death^25^, even though the majority of cells remains alive, especially at early time points after stimulation^11^,^25^, likely accounting for the upregulation in the *Nos2* gene. Importantly, the pharmacological inhibition of NO production in response to *S. typhimurium* and cytosolic flagellin resulted in a significant increase in the CFU numbers recovered from the macrophage cultures without inhibiting cell death. Therefore, even if it remains to be elucidated the relative contribution of the events induced by cytosolic flagellin in a single cell, our results strongly suggest that NOS2 activation and pyroptosis are independent effector mechanisms induced by NLRC4 inflammasomes that contribute to control intracellular infections.

Both *Nos2* and inflammasome activation are considered double-edged sword arms of immune responses, since in addition to their role to control infections, the overactivation of both mechanisms can cause tissue damage and consequent inflammatory pathologies such as asthma, cardiovascular and neurological diseases, liver and renal dysfunctions, atherosclerosis, tumors, coagulation disorders, sepsis, among others^8^,^33^,^34^,^35^. Therefore, the description of a novel role for caspase-1 on the epigenetic regulation of gene transcription could open up new perspectives in the therapeutic interventions for inflammatory disorders caused by uncontrolled activation of inflammasomes.

## Methods

### Mice and Cell Isolation

WT, casp-1/11^−/−^, NLRC4^−/−^ and transgenic mice (uncleavable PARP1 (PARP1^D214N^)) were bred from the same genetic background (C57BL/6) in our animal facilities at Federal University of São Paulo or the University of Zurich and maintained in a specific pathogen-free facility. All animal experiments were carried out in accordance with the Swiss, EU and Brazil ethical guidelines and have been approved by the local animal experimentation committee of the Canton of Zurich, Federal University of São Paulo and University of São Paulo under licenses #2012207, 0159–11 and 109/51-2, respectively, following the 3R guidelines. For the isolation of peritoneal macrophages (PMs), 9- to 12-week-old mice were i.p injected with 2 ml of 1,5% Starch solution (Sigma) from potatoes for 4 days. Mice were euthanized, cells were collected in cold PBS by abdominal lavage, and seeded in tissue culture plates (Costar) in full-RPMI medium containing 3% FCS, 100 units/ml penicillin/streptomycin, 1 mM sodium pyruvate and 2 mM _L_-glutamine and incubated at 37 °C and 5% CO_2_. All supplements were purchased from Invitrogen. The non-adherent cells were removed by vigorous washes with RPMI medium.

For the generation of bone marrow-derived macrophages (BMDM), 9–12 week-old mice were euthanized, and cells isolated as described elsewhere^46^. Briefly, cells were plated in 10 cm uncharged plastic plates (Petri dish) and incubated for full differentiation in RPMI medium supplemented with 10% FCS, 100 units/ml penicillin/streptomycin, 1 mM sodium pyruvate, 2 mM _L_-glutamine, 50 mM b-mercaptoethanol, and 20% L929 conditioned medium for 7 days in a humidified incubator (with 5% CO_2_ at 37 °C). After 7 days, cells were counted and seeded in 24-well tissue culture plates. After 16 hr, cells were stimulated as indicate.

### Stimulation of macrophages with flagellin

PM (3 × 10^5^) or BMDM (5 × 10^5^) were stimulated with purified flagellin from *Bacillus subtilis* or *S. Typhimurium* (Invivogen) [1–3 μg/ml] in its free form (FLA) or inserted into DOTAP (Dot) (Roche Diagnostics) (FLADot), a cationic lipid vesicle formulation that permits its delivery to cell cytosol and was used accordingly to the manufacturer’s instructions. Briefly, DOTAP (50 μl) was incubated for 15 min in serum-free media with 9 μg of purified flagellin. After incubation, 2.95 ml of RPMI 1640 medium was added, and an aliquot of 200 μl was added to 3 × 10^5^ macrophages (for 3 μg/ml final concentration).

### Bacterial Infection

Flagellin-suficient *S.typhimurium* 1412 strain (WT) or flagellin-deficient *S. typhimurium* 2157 strain (ΔFliC) were cultured overnight in L-broth at 37 °C with shaking. Then, bacteria were diluted 1:20 into L-broth, grown at 37 °C with shaking for 3 h, washed and resuspended in 0.9% NaCl saline before infection. Adherent PMs from C57BL/6 or casp1/11^−/−^ mice were infected at 1:10 multiplicity of infection (MOI) in antibiotic-free supplemented RPMI medium, in the presence or absence of 1 mM aminoguanidine (AG), a selective NOS2 inhibitor, centrifuged at 2000 rpm × 10 min to synchronize the infection and allowed to invade for 1 hr in the CO_2_ incubator. After infection, cultures were treated with gentamicin [50 μg/ml], in the presence or absence of AG, for 50 min to eliminate extracellular bacteria. Next, cultures were maintained with 5 μg/ml of gentamicin in the presence or absence of AG and purified *S. typhimurium* flagellin inserted into DOTAP (FliDot) [1 μg/mL], for 24–48 hr.

### Cytokine and Nitric Oxide measurement

IL-1β was measured in culture supernatants by enzyme-linked immunoabsorbent assay (ELISA) (kits from BD Biosciences) following the manufacturer’s instructions. Culture supernatants of *S. Typhimurium*-infected PMs were assayed for nitric oxide by the Griess reaction. Briefly, 50 μl of supernatant was incubated with 50 μl of Griess reagent for 5 min at room temperature. Nitrite concentration was determined by measuring the optical density at 550 nm in reference to a standard sodium nitrite solution.

### Western Blotting

Western blot was performed as previously described^47^. Cells were harvested, washed once in ice-cold PBS, lysed directly in SDS sample buffer (50 mMTris-HCl, pH 6.8, 2% SDS, 10% glycerol, and 2.5% L-mercaptoethanol), and boiled for 5 min. Samples were resolved under reducing conditions for 2–4 h at 100–120 V in SDS-polyacrylamide gels, according to its protein size. Proteins were then transferred onto PVDF membranes in a semi-dry system. Blots were blocked for 1 h in TBST (10 mMTris-HCl, pH 7.4, 150 mMNaCl, and 0.05% Tween) containing 0.1% sodium azide and 5% nonfat dried milk and then probed with polyclonal or monoclonal antibodies. Reactions were detected with suitable secondary antibody conjugated to horseradish peroxidase (The Jackson Laboratory and Amersham Biosciences) using enhanced chemiluminescence solution (Pierce).

### Cell cytotoxicity assay

Cell cytotoxicity was assessed using ethidium bromide (EtBr) incorporation in combination with acridine orange (AO) (Sigma) staining as described previously^25^. AO is a vital dye while EtBr is only incorporated by cells that lost membrane integrity. After 6–24 hr of stimulation or infection, the culture supernatants were removed, and a solution of PBS containing EtBr and AO (25 ng/mL; vol/vol) was added to cells. Images were acquired using an inverted fluorescence microscope with original magnification of 200 × . The percentage of EtBr single positive cells, representing the percentage of cell death, was analyzed using NIS-Elements Microscope (NIKON) software.

### RNA Extraction and Gene Expression Analysis by Real-Time RT-PCR

Confluent (90%) BMDM were stimulated with 3 μg/ml of Dot or FLADot for 3–4 hr. Total RNA was isolated using kits according to the manufacturers’ recommendation (Macherey-Nagel and Ambion). RNA was reverse-transcribed (kit from Applied Biosystems) and real-time PCR was performed using the Rotor-Gene 3000 (Corbett Life Science/QIAGEN) and TaqMan assays or SYBR Green.

### Chromatin Accessibility by ATAC-qPCR

Adherent PMs from C57BL/6 or casp1/11^−/−^ mice from each biological replicate were used to perform ATAC (assay for transposase-accessible chromatin) as previously described^3^. ATAC was performed on 5 × 10^5^ cells with Nextera DNA Sample Preparation Kit (FC-121-1030, Illumina). Total DNA was extracted and used as input. Validation of enrichment of open chromatin in the ATAC samples over input was performed by qPCR, testing the open chromatin genes TARS and beta-actin, and the closed chromatin markers RhO and CHEK2. Four primer sets covering the promoter region of *Nos2* was used to investigate the chromatin accessibility before and after flagellin infection. The primers used for ATAC-qPCR are described in Table S1.

## Additional Information

**How to cite this article**: Buzzo, C. L. *et al*. Epigenetic regulation of nitric oxide synthase 2, inducible (*Nos2*) by NLRC4 inflammasomes involves PARP1 cleavage. *Sci. Rep.* **7**, 41686; doi: 10.1038/srep41686 (2017).

**Publisher's note:** Springer Nature remains neutral with regard to jurisdictional claims in published maps and institutional affiliations.

## Supplementary Material

Supplemental Data

## References

[b1] Martinon F., Burns K. & Tschopp J. The inflammasome: a molecular platform triggering activation of inflammatory caspases and processing of proIL-beta. Molecular cell 10, 417–426 (2002). 12191486 10.1016/s1097-2765(02)00599-3

[b2] Broz P. & Dixit V. M. Inflammasomes: mechanism of assembly, regulation and signalling. Nat Rev Immunol. 16(7), 407–20 (2016). 27291964 10.1038/nri.2016.58

[b3] Gallo M. . MLL5 Orchestrates a Cancer Self-Renewal State by Repressing the Histone Variant H3.3 and Globally Reorganizing Chromatin. Cancer Cell 28, 715–729 (2015). 26626085 10.1016/j.ccell.2015.10.005

[b4] Saavedra P. H., Demon D., Van Gorp H. & Lamkanfi M. Protective and detrimental roles of inflammasomes in disease. Seminars in immunopathology 37, 313–322 (2015). 25895577 10.1007/s00281-015-0485-5

[b5] Jorgensen I. & Miao E. A. Pyroptotic cell death defends against intracellular pathogens. Immunol Rev 265, 130–142 (2015). 25879289 10.1111/imr.12287 PMC4400865

[b6] Lage S. L. . Emerging Concepts about NAIP/NLRC4 Inflammasomes. Front Immunol 5, 309 (2014). 25071770 10.3389/fimmu.2014.00309 PMC4078251

[b7] Bogdan C. Nitric oxide synthase in innate and adaptive immunity: an update. Trends Immunol 36, 161–178 (2015). 25687683 10.1016/j.it.2015.01.003

[b8] Pacher P., Beckman J. S. & Liaudet L. Nitric oxide and peroxynitrite in health and disease. Physiol Rev 87, 315–424 (2007). 17237348 10.1152/physrev.00029.2006 PMC2248324

[b9] Cheng H. . Nitric oxide in cancer metastasis. Cancer Lett 353, 1–7 (2014). 25079686 10.1016/j.canlet.2014.07.014 PMC4150837

[b10] Garry P. S., Ezra M., Rowland M. J., Westbrook J. & Pattinson K. T. The role of the nitric oxide pathway in brain injury and its treatment–from bench to bedside. Exp Neurol 263, 235–243 (2015). 25447937 10.1016/j.expneurol.2014.10.017

[b11] Buzzo C. L. . A novel pathway for inducible nitric-oxide synthase activation through inflammasomes. The Journal of biological chemistry 285, 32087–32095 (2010). 20702413 10.1074/jbc.M110.124297 PMC2952210

[b12] Goncalves V. M. . NLRP3 Controls Trypanosoma cruzi Infection through a Caspase-1-Dependent IL-1R-Independent NO Production. PLoS Negl Trop Dis 7, e2469 (2013). 24098823 10.1371/journal.pntd.0002469 PMC3789781

[b13] Hecker M., Cattaruzza M. & Wagner A. H. Regulation of inducible nitric oxide synthase gene expression in vascular smooth muscle cells. Gen Pharmacol 32, 9–16 (1999). 9888247 10.1016/s0306-3623(98)00082-2

[b14] Kleinert H., Euchenhofer C., Ihrig-Biedert I. & Forstermann U. In murine 3T3 fibroblasts, different second messenger pathways resulting in the induction of NO synthase II (iNOS) converge in the activation of transcription factor NF-kappaB. The Journal of biological chemistry 271, 6039–6044 (1996). 8626388 10.1074/jbc.271.11.6039

[b15] Kleinert H. . Cytokine induction of NO synthase II in human DLD-1 cells: roles of the JAK-STAT, AP-1 and NF-kappaB-signaling pathways. Br J Pharmacol 125, 193–201 (1998). 9776360 10.1038/sj.bjp.0702039 PMC1565595

[b16] Marks-Konczalik J., Chu S. C. & Moss J. Cytokine-mediated transcriptional induction of the human inducible nitric oxide synthase gene requires both activator protein 1 and nuclear factor kappaB-binding sites. The Journal of biological chemistry 273, 22201–22208 (1998). 9712833 10.1074/jbc.273.35.22201

[b17] Aktan F. iNOS-mediated nitric oxide production and its regulation. Life Sci 75, 639–653 (2004). 15172174 10.1016/j.lfs.2003.10.042

[b18] Deguine J. & Barton G. M. MyD88: a central player in innate immune signaling. F1000Prime Rep 6, 97 (2014). 25580251 10.12703/P6-97 PMC4229726

[b19] Narayanan K. B. & Park H. H. Toll/interleukin-1 receptor (TIR) domain-mediated cellular signaling pathways. Apoptosis 20, 196–209 (2015). 25563856 10.1007/s10495-014-1073-1

[b20] Bogdan C. Nitric oxide and the regulation of gene expression. Trends Cell Biol 11, 66–75 (2001). 11166214 10.1016/s0962-8924(00)01900-0

[b21] Kamijo R. . Requirement for transcription factor IRF-1 in NO synthase induction in macrophages. Science (New York, N.Y) 263, 1612–1615 (1994). 10.1126/science.7510419 7510419

[b22] Lowenstein C. J. . Macrophage nitric oxide synthase gene: two upstream regions mediate induction by interferon gamma and lipopolysaccharide. Proceedings of the National Academy of Sciences of the United States of America 90, 9730–9734 (1993). 7692452 10.1073/pnas.90.20.9730 PMC47644

[b23] Kim Y. I., Park S. W., Kang I. J., Shin M. K. & Lee M. H. Activin suppresses LPS-induced Toll-like receptor, cytokine and inducible nitric oxide synthase expression in normal human melanocytes by inhibiting NF-kappaB and MAPK pathway activation. Int J Mol Med 36, 1165–1172 (2015). 26259928 10.3892/ijmm.2015.2308

[b24] Cuzzocrea S. . Pyrrolidine dithiocarbamate attenuates the development of acute and chronic inflammation. Br J Pharmacol 135, 496–510 (2002). 11815386 10.1038/sj.bjp.0704463 PMC1573136

[b25] Lage S. L. . Cytosolic flagellin-induced lysosomal pathway regulates inflammasome-dependent and -independent macrophage responses. Proceedings of the National Academy of Sciences of the United States of America 110, E3321–3330 (2013). 23942123 10.1073/pnas.1305316110 PMC3761566

[b26] Erener S. . Inflammasome-Activated Caspase 7 Cleaves PARP1 to Enhance the Expression of a Subset of NF-kappaB Target Genes. Molecular cell 46, 200–211 (2012). 22464733 10.1016/j.molcel.2012.02.016

[b27] Garber M. . A high-throughput chromatin immunoprecipitation approach reveals principles of dynamic gene regulation in mammals. Molecular cell 47, 810–822 (2012). 22940246 10.1016/j.molcel.2012.07.030 PMC3873101

[b28] Lupfer C., Malik A. & Kanneganti T. D. Inflammasome control of viral infection. Curr Opin Virol 12, 38–46 (2015). 25771504 10.1016/j.coviro.2015.02.007 PMC4470791

[b29] Ulland T. K., Ferguson P. J. & Sutterwala F. S. Evasion of inflammasome activation by microbial pathogens. The Journal of clinical investigation 125, 469–477 (2015). 25642707 10.1172/JCI75254 PMC4319426

[b30] van de Veerdonk F. L., Joosten L. A. & Netea M. G. The interplay between inflammasome activation and antifungal host defense. Immunol Rev 265, 172–180 (2015). 25879292 10.1111/imr.12280

[b31] Zamboni D. S. & Lima-Junior D. S. Inflammasomes in host response to protozoan parasites. Immunol Rev 265, 156–171 (2015). 25879291 10.1111/imr.12291

[b32] Lima-Junior D. S. . Inflammasome-derived IL-1beta production induces nitric oxide-mediated resistance to Leishmania. Nat Med 19, 909–915 (2013). 23749230 10.1038/nm.3221

[b33] Ghasemi M. & Fatemi A. Pathologic role of glial nitric oxide in adult and pediatric neuroinflammatory diseases. Neurosci Biobehav Rev 45, 168–182 (2014). 24933692 10.1016/j.neubiorev.2014.06.002

[b34] Ten Broeke R. . Overexpression of endothelial nitric oxide synthase suppresses features of allergic asthma in mice. Respir Res 7, 58 (2006). 16597326 10.1186/1465-9921-7-58 PMC1456969

[b35] Xu C., Yi C., Wang H., Bruce I. C. & Xia Q. Mitochondrial nitric oxide synthase participates in septic shock myocardial depression by nitric oxide overproduction and mitochondrial permeability transition pore opening. Shock 37, 110–115 (2012). 21993446 10.1097/SHK.0b013e3182391831

[b36] Mangge H., Becker K., Fuchs D. & Gostner J. M. Antioxidants, inflammation and cardiovascular disease. World J Cardiol 6, 462–477 (2014). 24976919 10.4330/wjc.v6.i6.462 PMC4072837

[b37] Dupont L. L., Glynos C., Bracke K. R., Brouckaert P. & Brusselle G. G. Role of the nitric oxide-soluble guanylyl cyclase pathway in obstructive airway diseases. Pulm Pharmacol Ther 29, 1–6 (2014). 25043200 10.1016/j.pupt.2014.07.004

[b38] Malireddi R. K., Ippagunta S., Lamkanfi M. & Kanneganti T. D. Cutting edge: proteolytic inactivation of poly(ADP-ribose) polymerase 1 by the Nlrp3 and Nlrc4 inflammasomes. J Immunol 185, 3127–3130 (2010). 20713892 10.4049/jimmunol.1001512 PMC3104018

[b39] Hassa P. O., Haenni S. S., Elser M. & Hottiger M. O. Nuclear ADP-ribosylation reactions in mammalian cells: where are we today and where are we going? Microbiol Mol Biol Rev 70, 789–829 (2006). 16959969 10.1128/MMBR.00040-05 PMC1594587

[b40] Kim M. Y., Zhang T. & Kraus W. L. Poly(ADP-ribosyl) ation by PARP-1: ‘PAR-laying’ NAD+into a nuclear signal. Genes Dev 19, 1951–1967 (2005). 16140981 10.1101/gad.1331805

[b41] D’Amours D., Sallmann F. R., Dixit V. M. & Poirier G. G. Gain-of-function of poly(ADP-ribose) polymerase-1 upon cleavage by apoptotic proteases: implications for apoptosis. J Cell Sci 114, 3771–3778 (2001). 11707529 10.1242/jcs.114.20.3771

[b42] Kraus W. L. & Lis J. T. PARP goes transcription. Cell 113, 677–683 (2003). 12809599 10.1016/s0092-8674(03)00433-1

[b43] Krishnakumar R. & Kraus W. L. PARP-1 regulates chromatin structure and transcription through a KDM5B-dependent pathway. Molecular cell 39, 736–749 (2010). 20832725 10.1016/j.molcel.2010.08.014 PMC2939044

[b44] Petrilli V. . Noncleavable poly(ADP-ribose) polymerase-1 regulates the inflammation response in mice. The Journal of clinical investigation 114, 1072–1081 (2004). 15489954 10.1172/JCI21854 PMC522248

[b45] Hottiger M. O. Poly(ADP-ribose) polymerase inhibitor therapeutic effect: are we just scratching the surface? Expert Opin Ther Targets 19, 1149–1152 (2015). 26212149 10.1517/14728222.2015.1073262

[b46] Petrilli V. . Activation of the NALP3 inflammasome is triggered by low intracellular potassium concentration. Cell death and differentiation 14, 1583–1589 (2007). 17599094 10.1038/sj.cdd.4402195

[b47] Weinlich R. . TLR4/MYD88-dependent, LPS-induced synthesis of PGE2 by macrophages or dendritic cells prevents anti-CD3-mediated CD95L upregulation in T cells. Cell death and differentiation 15, 1901–1909 (2008). 18820644 10.1038/cdd.2008.128

