# Supplementary material for: Epigenetic regulation of nitric oxide synthase 2, inducible (Nos2) by NLRC4 inflammasomes involves PARP1 cleavage
Source: Sci Rep. 2017 Feb 2;7:41686. doi: 10.1038/srep41686 (PMC5288713; doi:10.1038/srep41686)
Supplement: Supplemental Data [file srep41686-s1.pdf]

Epigenetic regulation of nitric oxide synthase 2, inducible (*Nos2*) by NLRC4 inflammasomes involves PARP1 cleavage

Carina de Lima Buzzo [1,2]; Tiago Medina[3]; Laura M. Branco[1,4], Silvia L. Lage[1,4], Luís Carlos de Souza Ferreira[2], Gustavo P. Amarante-Mendes[4]; Michael O. Hottiger[5], Daniel D. De Carvalho[3,6], Karina R. Bortoluci[1]

[1]Centro de Terapia Celular e Molecular (CTC-Mol) e Departamento de Ciências Biológicas - Universidade Federal de São Paulo, São Paulo, Brazil.
[2]Departamento de Microbiologia, Instituto de Ciências Biomédicas, Universidade de São Paulo, São Paulo, Brazil.
[3]Princess Margaret Cancer Centre, University Health Network, Toronto, ON, M5G 2M9, Canada.
[4]Instituto de Ciências Biomédicas, Universidade de São Paulo, São Paulo and Instituto de Investigação em Imunologia, Instituto Nacional de Ciência e Tecnologia (INCT-iii), Brazil.
[5]Department of Molecular Mechanisms of Disease, University of Zurich, Zurich, Switzerland.
[6]Department of Medical Biophysics, University of Toronto, Toronto, ON, M5G 2M9, Canada

Locus	Primer sequence (5’-3’)	Amplicon length (bp)
Nos2 (Primer set 1)		
Forward	GGG TGT TGC CTG GAT AAA GA	78
Reverse	CAC TTG CAC ACA CAC ACA GC	
Nos2 (Primer set 2)		
Forward	ATG GCC TTG CAT GAG GAT AC	172
Reverse	GCA GCA GCC ATC AGG TAT TT	
Nos2 (Primer set 3)		
Forward	CCA TGC CCA GCT TTT GAA CT	81
Reverse	TAA TTG GGC TGG GGA GAT GG	
Nos2 (Primer set 4)		
Forward	AGC CTA GTC AAC TGC AAG GT	87
Reverse	CCT GGT TTT CTG GGC TTT CC	
TARS (Positive Control)		
Forward	GGC GGT GTA GAC CTA CAA TTC C	183
Reverse	AGG GTT CGT CAT TCT CGG CTA T	
β-actin (Positive Control)		
Forward	CGG CAA CGA AGG AGC TGC AAA	138
Reverse	GCC CGC CGT TCC GAA AGT TG	
RhO (Negative Control)		
Forward	CCA GTG TCA CCG TCC TCA GA	148
Reverse	TCC CTG GGA ATG CTG CAC TT	
ChEK2 (Negative Control)		
Forward	CCC CTG ATG CTG TCC ACT GT	127
Reverse	GGT TGC CGT ATC CCT GAA GAG A	

Supplemental Table - Primers used for ATAC-qPCR assay.

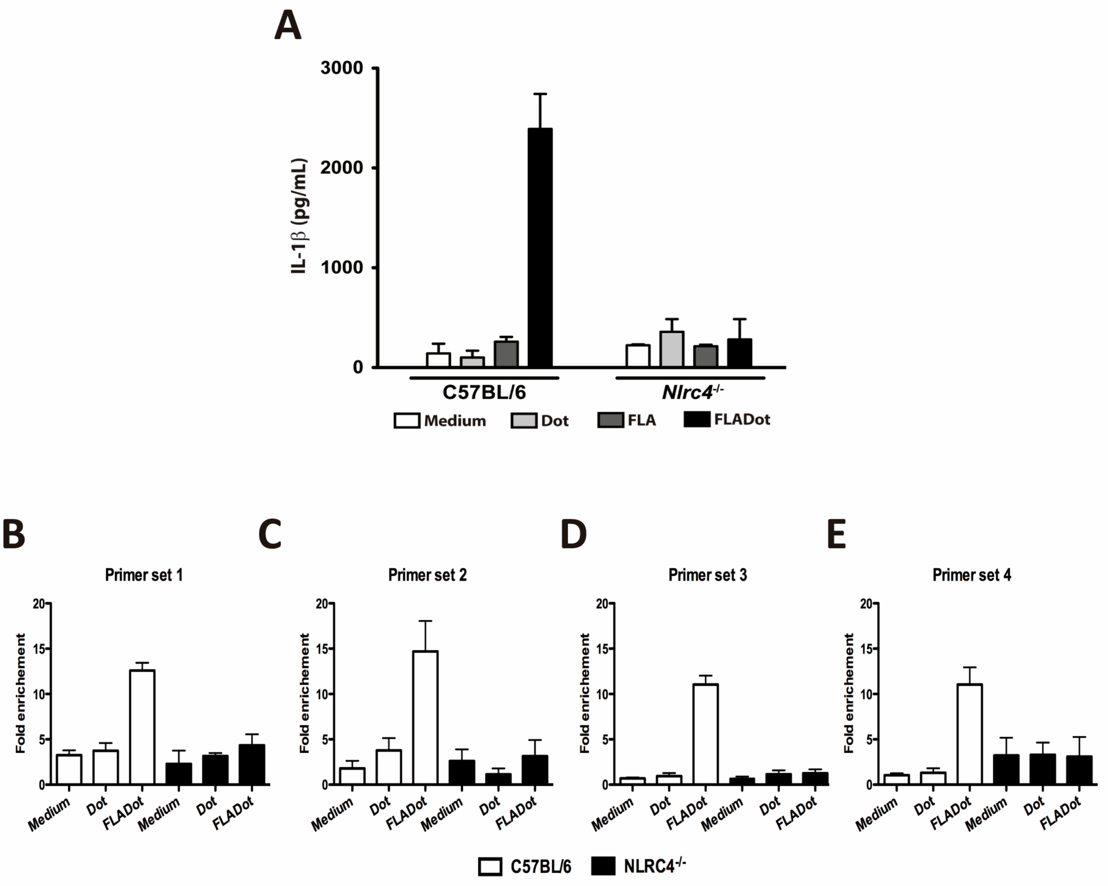

Supplemental Figure 1: Binding sites of NF-κB at the *iNOS* gene promoter are not accessible in the absence of NLRC4. (A) PMs isolated from C57BL/6 WT and NLRC4$^{-/-}$ mice were stimulated with FLA or FLADot [3μg/mL] for 6 hr and IL-1β secretion was analyzed by ELISA in the culture supernatant. Bars represent the mean ± SEM of triplicate samples. Data are representative of three independent experiments. **(B)** BMDM from WT or NLRC4$^{-/-}$ were stimulated with Dot or FLADot and the chromatin accessibility at the *iNOS* gene promoter was analyzed using Transpoase-Accessible Chromatin Assay (ATAC) coupled with qPCR.
